# Whole-Genome Sequencing for High-Consequence Emerging RNA Viruses: Strategy Selection for Bundibugyo Virus Disease Under 2026 Outbreak Constraints

**DOI:** 10.3390/v18070714

**Published:** 2026-06-28

**Authors:** Katharina Kopp

**Affiliations:** One Health Pathogenomics Consulting, 80687 Munich, Germany; koppk@onehealth-pathogenomics-consulting.org

**Keywords:** *Orthoebolavirus bundibugyoense*, Bundibugyo virus disease, filoviruses, whole-genome sequencing, outbreak genomics, random-primed cDNA sequencing, target enrichment, probe-based hybrid capture, tiled amplicon sequencing, genomic epidemiology

## Abstract

Whole-genome sequencing (WGS) is central to outbreak response for high-consequence ribonucleic acid (RNA) viruses, but useful genomes depend on workflow design, sample quality, biosafety, diagnostic breadth, infrastructure, and bioinformatics as much as sequencing platform. The 2026 Bundibugyo virus disease outbreak in the Democratic Republic of the Congo and Uganda provides a case example. Bundibugyo virus (BDBV) was already known from the 2007–2008 Uganda and 2012 Democratic Republic of the Congo outbreaks, but sparse historical genome sampling, Ebola virus-centered diagnostic assumptions, non-specific febrile and viral hemorrhagic fever presentations, and difficult field conditions created a need for broad differential diagnosis, rapid species assignment, and representative genome generation. This review compares outbreak WGS strategies by the degree of prior viral sequence knowledge required, distinguishing direct RNA sequencing, random-primed complementary DNA (cDNA) sequencing, sequence-independent amplified cDNA sequencing, background-depleted or particle-enriched cDNA sequencing, probe-based hybrid capture, tiled amplicon sequencing, portable field sequencing, culture-derived sequencing, and associated bioinformatics workflows. For BDBV-like outbreaks, the most defensible strategy is staged and adaptive: broad viral hemorrhagic fever and febrile illness differential testing at recognition; Filoviridae-wide testing when filovirus disease remains plausible; divergence-tolerant first-genome recovery; quantification-cycle-informed sequencing prioritization without BDBV-only diagnostic narrowing; validated amplicon scale-up; and representative sequencing across locations and time.

## 1. Introduction

Whole-genome sequencing (WGS) has become a central tool in outbreak response for RNA viruses. Genome data can confirm virus identity, support species assignment, monitor diagnostic targets, reconstruct transmission, estimate outbreak timing, track geographic spread, and identify sequence features relevant to vaccines, antibodies, and antivirals. This has been shown specifically during outbreaks caused by BDBV [1,2,3,4,5,6] and Ebola virus (EBOV, species *Orthoebolavirus zairense*) [7,8,9]. However, WGS is not selected by sequencing platform alone. During outbreaks, the most important question is often not which technology gives the most complete genome under ideal conditions, but which workflow can generate interpretable genomes quickly, safely, locally, and representatively enough to inform response.

The practical constraints are substantial. Viral load, sample type, host background, RNA degradation, transport delay, diagnostic uncertainty, biosafety requirements, reagent supply, local sequencing access, laboratory infrastructure, staff training, contamination control, and bioinformatics capacity all determine whether genome generation is feasible [10,11,12,13,14,15,16,17,18,19,20,21,22,23,24,25,26]. A method that works well in a high-resource reference laboratory may be inappropriate in a remote or conflict-affected outbreak region. Conversely, a rapid field workflow may be unsuitable if it depends on primers or panels that have not been checked against the outbreak virus.

The 2026 Bundibugyo virus disease (BVD) epidemic makes BDBV, assigned to the species *Orthoebolavirus bundibugyoense*, a high-stakes case example for evaluating WGS strategy selection under diagnostic, genomic, biosafety, and infrastructure constraints. This human-pathogenic ebolavirus species was first recognized during the 2007–2008 outbreak in Uganda and later caused an outbreak in the Democratic Republic of the Congo (DRC) in 2012 [1,2,3,4]. The virus was therefore known before the 2026 outbreak, but it remained far less genomically represented than EBOV or severe acute respiratory syndrome coronavirus 2 (SARS-CoV-2). The initial 2026 genome report analyzed three newly generated genomes in the context of 34 historical BDBV genomes from previous outbreaks [6]. This creates a distinct outbreak genomics problem: BDBV is not an unknown pathogen, but neither is it a mature surveillance target with dense genomic baselines, validated primer schemes, and extensive countermeasure evidence.

This review synthesizes and critically evaluates WGS workflows for RNA virus outbreaks under operational constraints, using the 2026 BVD outbreak as a case example. Evidence from BDBV, EBOV, SARS-CoV-2, chikungunya virus (CHIKV), dengue virus (DENV), respiratory virus sequencing, wastewater surveillance, mpox virus, inactivation studies, viral capture systems, random-primed cDNA sequencing, and sequence-independent amplification methods is used selectively to identify transferable workflow principles. The aim is not to rank platforms, but to clarify how diagnostic breadth, reverse transcription priming, wet laboratory target dependence, viral input enrichment, validated inactivation, local implementation, sampling representativeness, and downstream bioinformatics should shape WGS decisions during BDBV-like outbreaks.

## 2. The 2026 Bundibugyo Virus Disease Outbreak as a Case Example

The first BDBV outbreak in Uganda in 2007/2008 already demonstrated the diagnostic and genomic problem created by a divergent but related ebolavirus. Molecular assays directed toward then-recognized filoviruses did not initially identify the causative agent. Evidence of acute ebolavirus infection was detected by broader antigen and serological testing, while highly sensitive quantitative reverse transcription polymerase chain reaction (RT-qPCR) assays specific for known Zaire and Sudan ebolaviruses and marburgviruses were initially negative [1]. A broadly reactive filovirus large polymerase gene (L-gene) reverse transcription polymerase chain reaction (RT-PCR) yielded a sequenceable amplicon, and random-primed pyrosequencing rapidly generated more than 70% of the viral genome from total RNA extracted from patient serum [1]. The draft sequence enabled development of a BDBV-specific nucleoprotein (NP) RT-qPCR assay, and the complete genome was finished using a classic primer-walking strategy from the reference virus isolate [1]. Thus, the first BDBV genome was not produced by a mature BDBV-specific tiling scheme, but by diagnostic escalation, random-primed sequencing, and primer walking after partial sequence recovery.

The 2026 BVD outbreak made this issue operational in a setting where the initial clinical syndrome was not specific for any single filovirus. The outbreak was officially reported as Ebola disease caused by Bundibugyo virus in the DRC and Uganda [5], and the initial public genome report described testing of clinical specimens at the Institut National de Recherche Biomédicale (INRB) in Kinshasa using Xpert Ebola Assay/GeneXpert system (Cepheid, Sunnyvale, CA, USA), RADIONE Pan-Ebola Genotyping & Marburg Multiplex Kit/RADIONE system (KH Medical, Pyeongtaek-si, Republic of Korea), and the RealStar Filovirus Screen RT-PCR Kit 1.0 (altona Diagnostics GmbH, Hamburg, Germany) [6]. The Xpert Ebola Assay/GeneXpert system is EBOV-focused, and all tested specimens were negative with the Xpert Ebola Assay/GeneXpert system, whereas eight of thirteen RNA samples were positive with the RealStar Filovirus Screen RT-PCR Kit 1.0 [6]. This pattern is best interpreted as a target scope mismatch of the EBOV-focused Xpert Ebola Assay/GeneXpert system rather than as a failure of near-patient molecular diagnostics as a platform. Broad filovirus RT-PCR assays serve a different diagnostic purpose because they are designed to detect multiple filovirus species or broader filovirus diversity, although their performance still depends on assay design, target coverage, validation material, and compatibility with the outbreak virus [27,28,29,30,31,32]. The Xpert Ebola Assay remains valuable when EBOV is the diagnostic target, but negative results with this EBOV-focused assay cannot exclude BDBV, Sudan virus, Marburg virus, or other non-EBOV filoviruses [33,34]. At the same time, a front-end outbreak investigation should not be narrowed to pan-filovirus testing alone. Crimean–Congo hemorrhagic fever (CCHF) has been detected during Uganda filovirus outbreak response [35]. Bas-Congo virus (BASV) illustrates that some viral hemorrhagic fever (VHF)-like clusters may require discovery sequencing because no existing panel or PCR assay would detect the previously unknown causative agent [36]. Severe malaria, often suddenly occurring in clusters of children, can produce high fever and dark urine that may mimic hemorrhagic disease [37]. Sosuga virus provides another non-filovirus example in which deep sequencing and metagenomic analysis identified a novel paramyxovirus after standard testing for suspected pathogens had been negative [38].

The 2026 outbreak preliminary WGS report illustrates current genome recovery practice for BDBV [6]. In the DRC workflow, RNA was extracted from inactivated specimens, reverse transcribed, prepared using Illumina RNA Prep with Enrichment (Illumina, Inc., San Diego, CA, USA) and the Twist Comprehensive Viral Research Panel (Twist Bioscience, South San Francisco, CA, USA), and sequenced on a NextSeq system (Illumina, Inc., San Diego, CA, USA) and a GridION sequencer (Oxford Nanopore Technologies plc, Oxford, UK). Two near-complete genomes were obtained in less than 16 h from sample reception [6]. In Uganda, a probe-based hybrid capture workflow using the Illumina Viral Surveillance Panel (Illumina, Inc., San Diego, CA, USA) and NextSeq 2000 system (Illumina, Inc., San Diego, CA, USA) recovered approximately 99% genome coverage from an imported fatal case at approximately 100× average depth [6]. These workflows are best described as probe-based target enrichment workflows rather than metagenomic workflows. They show why hybrid capture is attractive when the virus is known at family, genus, or species level but insufficiently sampled for immediate reliance on highly optimized tiled amplicon schemes.

The 2026 genome updates also demonstrate a major interpretation constraint: early genome sets are rarely representative. Additional genomes became available from Bunia, Katwa, Hoho, Lumumba, and ex-Bunia cases, but the update noted that all but one genome still came from Bunia or ex-Bunia, so time to most recent common ancestor (tMRCA) estimates were interpreted as applying mainly to the Bunia epidemic rather than to the full geographic range of recorded cases [6]. This point is central. A few early genomes can confirm species identity and local lineage structure, but they cannot fully resolve phylogeography, number of introductions, or unsampled transmission chains across all affected locations. The historical BDBV genome dataset also requires careful interpretation: the 2007–2008 National Center for Biotechnology Information (NCBI) records include multiple entries for a small number of named isolates, and the 2012 DRC dataset includes both initially sequenced patient genomes and later retrospective sequences, some of which appear to represent the same isolate name after different sequencing, isolation, or passage histories [1,2,3]. Genome-based outbreak reconstruction should therefore explicitly dereplicate technical replicates, culture-derived sequences, and repeated isolate submissions before inference.

## 3. What Whole-Genome Sequencing Must Deliver During an Outbreak

During an outbreak, WGS may serve several complementary purposes, including species assignment, diagnostic target assessment, phylogenetic reconstruction, phylogeographic inference, countermeasure evaluation, and monitoring of viral evolution. Each application places different demands on sample selection, sequencing depth, genome completeness, metadata quality, and workflow design.

First, WGS supports species assignment when pan-filovirus or VHF panel testing [27,28,29,30,31,32] indicates a possible high-consequence viral infection but species identity remains unresolved, and it also supports investigation when EBOV-specific assays [33,34] are negative but clinical and epidemiological suspicion remains high. This is particularly important because clinical presentation cannot reliably distinguish, for example EBOV, BDBV, Sudan virus, Marburg virus, Crimean–Congo hemorrhagic fever virus, severe malaria, or other tropical febrile illnesses and VHF-like syndromes at presentation [1,4,35,36,37,38].

Second, WGS supports diagnostic target assessment. During EBOV outbreak sequencing, genome sequences revealed mismatches with diagnostic PCR probe sequences [7]. For BDBV, new viral genome sequences are needed to determine whether primers, probes, capture panels, and tiled amplicon schemes remain compatible with the current outbreak virus [1,2,3,6,13,14,15,16,17,18,27,28,29,30,31,32].

Third, WGS informs planning and interpretation of potential medical countermeasures. For filoviruses, licensed vaccines and antibody-based therapeutics are currently limited to Ebola virus disease caused by EBOV, whereas no vaccine, monoclonal antibody, antiviral, or post-exposure prophylaxis is licensed specifically for BDBV disease as of 25 June 2026. However, BDBV-specific and potentially cross-reactive candidate medical countermeasures, including monoclonal antibodies, antivirals, post-exposure prophylaxis candidates, and vaccines, are under active consideration for outbreak-associated clinical evaluation during the 2026 BVD outbreak [39]. For BDBV and other ebolaviruses, glycoprotein (GP) monitoring is critical for monoclonal antibodies, vaccines, and entry-focused interventions [8,40,41,42,43], whereas polymerase and replication complex monitoring is relevant for therapeutic and post-exposure prophylaxis antiviral candidates such as nucleoside analogues. Whole-genome sequencing is therefore preferable to single-gene sequencing because different medical countermeasure classes depend on different viral targets and because mutations outside the primary target region may influence viral fitness, transmission, or interpretation of treatment-associated selection. Sequence data cannot by themselves prove antibody escape, vaccine failure, or antiviral resistance, but they can identify changes that require experimental or clinical follow-up before or during outbreak-associated evaluation of candidate medical countermeasures.

Fourth, WGS supports phylogenetic and phylogeographic inference. Dense, representative sampling can help distinguish local expansion from multiple introductions, detect cross-border movement, and estimate outbreak timing [2,3,6,7,8,9,44,45,46,47,48,49,50,51]. Sparse or geographically biased sampling can mislead these analyses, particularly when most genomes come from one referral center or outbreak focus [6].

Fifth, WGS can support decentralized response if genomes can be generated close to the outbreak. Field-deployable EBOV sequencing demonstrated during the 2013–2016 West African EBOV epidemic that near-real-time genomics is possible for high-consequence RNA viruses [17,52]. However, portable sequencing requires more than a sequencing device. It requires validated inactivation, nucleic acid extraction, library preparation, contamination control, cold-chain or reagent planning, bioinformatics, quality assurance, and metadata capture [17,52,53]. 

Broader SARS-CoV-2 genomic surveillance and assembly comparison experience also showed that platform choice, assembly strategy, quality control, and bioinformatics implementation affect the usability of genome outputs [54,55]. Table 1 summarizes the main outbreak questions that WGS can address and the corresponding sequencing requirements.

## 4. Determinants of Workflow Choice Under Outbreak Constraints

Workflow choice should not be reduced to a single continuum. The amount of prior viral sequence information influences primer/probe confidence and interpretation, but it is only one dimension. Other independent dimensions include reverse transcription priming strategy, viral input gain before sequencing, timing of concentration relative to validated inactivation, field feasibility, reagent dependence, turnaround time, sample throughput, tolerance of degraded or low-viral-load material, sequencing read type, and bioinformatics strategy. Culture-derived sequencing, for example, can provide high viral input without being intrinsically primer- or probe-dependent, whereas tiled amplicon sequencing is highly target design-dependent but operationally efficient once validated. Conversely, random-primed cDNA sequencing from total RNA can support genome recovery without any virus-specific primer or probe, but its efficiency depends strongly on viral load, host background, and sequencing depth.

The first is time pressure. During initial recognition, species assignment and diagnostic escalation may be more urgent than perfect genome completeness. During expansion, throughput and decentralization become more important. During retrospective reconstruction, genome completeness, sampling representativeness, and metadata quality dominate interpretation.

The second is sample type and viral load. Whole blood, serum, plasma, swabs, cerebrospinal fluid, tissue, and culture supernatant differ in host background, inhibitors, RNA stability, and viral abundance. Sequence-agnostic random-primed cDNA sequencing can work well for high-titer specimens but may yield incomplete genomes when host nucleic acid dominates [10,11,12,19,20].

The third is biosafety and inactivation status. For filoviruses, sequencing RNA extracted from validated inactivated material is fundamentally different from concentrating intact virions, isolating infectious virus, or purifying virus from culture [23,24,25,26,56,57,58,59,60]. Virus isolation followed by ultracentrifugation may produce high-input material, but for filoviruses it is a maximum containment (biosafety level 4, BSL-4) reference laboratory workflow, not a routine field strategy.

The fourth is local infrastructure. Ultracentrifuges, automated extraction instruments, cold-chain logistics, sequencers, clean work areas, stable electricity, trained personnel, and bioinformatics support cannot be assumed in remote or conflict-affected outbreak settings. Portable sequencing reduces some barriers but does not remove the need for a complete workflow [17,52,53].

The fifth is target dependence. WGS approaches can be broadly separated by how much viral sequence must be known before sequencing starts. Direct RNA sequencing reads native RNA but is not the routine outbreak WGS workflow. Most RNA virus WGS instead passes through reverse transcription (RT) to complementary DNA (cDNA). In the least target-dependent cDNA workflows, reverse transcription is random-primed, and the sequencing library is analyzed by taxonomic classification, reference-guided mapping, and/or *de novo* assembly. Sequence-independent amplification adds universal tag or random primer amplification without virus-specific primers. Probe-based hybrid capture is semi-targeted because it enriches cDNA through viral probes designed from known sequence diversity. Tiled amplicon sequencing is the most target-dependent WGS approach because primer pairs tile across a known genome. These approaches should therefore be selected according to the question being asked, not forced into a single hierarchy.

## 5. RNA Virus WGS Terminology: RT Priming, cDNA Enrichment, Amplification, and Assembly

For RNA viruses, “whole-genome sequencing” describes the intended output: recovery of a complete or near-complete viral genome. It does not define the molecular substrate that enters the sequencer. Except for direct RNA sequencing, most RNA virus WGS workflows first convert viral RNA into cDNA by reverse transcription, often followed by second-strand synthesis and standard library preparation. Thus, the decisive workflow questions are how reverse transcription is primed, whether the cDNA is enriched or amplified before sequencing, and whether reads are interpreted by reference-based mapping, *de novo* assembly, or both.

In this review, next-generation sequencing (NGS) refers to the sequencing technology, whereas WGS refers to the genome recovery goal. Direct RNA sequencing is the only category that reads native RNA without cDNA conversion, but it is not the default high-sensitivity outbreak WGS workflow. Routine RNA virus outbreak WGS more commonly sequences cDNA generated from total RNA, enriched viral particles, hybrid-captured targets, or tiled amplicons. The term RNA sequencing is therefore used here only when needed as a broad library description term, not to imply host transcriptome profiling.

The broadest routine cDNA-based approach is random-primed sequencing of cDNA generated from total RNA. This approach requires little or no prior viral sequence knowledge because reads can be classified taxonomically, mapped when a plausible reference exists, or assembled *de novo* when the agent is divergent or unknown. It is nevertheless not unbiased: extraction chemistry, depletion steps, random priming efficiency, reverse transcription performance, library preparation, sequencing platform, database composition, and analysis pipeline all affect genome recovery [10,11,12,19,20].

Sequence-independent amplification, including sequence-independent single-primer amplification (SISPA) approaches, is still sequence-agnostic with respect to the viral genome, but it adds amplification from universal or tagged random primers rather than virus-specific primers [21,22]. Probe-based hybrid capture is semi-targeted because viral cDNA is enriched using probes designed from known sequence diversity [13,14,18]. Tiled amplicon sequencing is highly targeted because primer pairs amplify overlapping regions of a known genome [15,16,17,53,61,62,63,64,65,66]. Targeted short-region Sanger sequencing can support species confirmation or genotyping but is not WGS unless the amplicons tile across the genome. Table 2 summarizes these categories by prior sequence knowledge and main bias-determining step.

Oligo-deoxythymidine (oligo-dT)-based transcriptome sequencing is not treated as a general viral genome WGS strategy in this review. It preferentially captures polyadenylated RNA and is therefore inappropriate as a broad WGS approach for filoviruses, arenaviruses, bunyaviruses, flaviviruses, and many other VHF or emerging zoonotic RNA virus contexts in which random priming, sequence-independent amplification, probe-based capture, or validated tiled amplicons are the relevant options.

## 6. Sequence-Agnostic and Sequence-Independent cDNA Sequencing

Sequence-agnostic random-primed cDNA sequencing is the broadest routine WGS approach for RNA virus outbreaks. It does not use virus-specific primers or probe panels before library preparation. Instead, RNA is extracted, reverse transcribed with random primers, converted into sequencing libraries, and analyzed by taxonomic classification, reference-guided mapping, and/or *de novo* assembly. This category is most useful when the causative agent is unknown, when targeted tests are negative or incomplete despite high clinical or epidemiological suspicion, or when pathogen discovery remains part of the outbreak question.

Enhanced random-primed sequencing workflows have generated complete *de novo* assemblies of Lassa virus and EBOV genomes from clinical and biological samples [19]. Published nanopore and Illumina workflows have also recovered CHIKV and DENV genomes directly from clinical material [20]. The original BDBV discovery is directly relevant: more than 70% of the viral genome was recovered by random-primed pyrosequencing from patient serum before the genome was completed by primer walking [1]. These examples show that sequence-agnostic cDNA sequencing can support genome recovery during outbreaks, but performance depends strongly on viral load, sample type, host background, sequencing depth, and bioinformatics support.

Host background reduction can improve sequence-agnostic cDNA sequencing by increasing the viral fraction without introducing virus-specific primers or probes. Approaches include ribosomal RNA depletion, methylated host deoxyribonucleic acid (DNA) depletion, deoxyribonuclease (DNase) treatment, clarification, filtration, and enrichment of particle-protected nucleic acids [10,11,19,20,23,24]. These steps may improve genome recovery but can also add cost, hands-on time, and workflow variability.

Sequence-independent amplification, including SISPA-like approaches, should be kept conceptually separate from both random-primed cDNA sequencing without amplification and target enrichment. Such methods use random or sequence-independent priming and amplification to increase low-input material, but they do not use virus-specific primers or probes [21,22]. They can support genome recovery when input is limited or sequence identity is uncertain, but they can introduce amplification bias, uneven coverage, chimeras, and unreliable low-frequency variant calls. This distinction matters because species confirmation, consensus genome generation, and intrahost variant analysis have different evidentiary thresholds.

For sequence-agnostic and sequence-independent amplified approaches, the bioinformatics workflow is central. As in other whole-viral-genome assembly workflows, read quality control and adapter trimming are required. Preliminary taxonomic classification can be used to assess potential contamination and, where appropriate, to guide host-read subtraction. This step can also indicate whether a plausible reference is available for reference-guided mapping and assembly. If the target is divergent or unknown, de novo assembly is required to reconstruct the viral genome [10,11,12,19,20].

Short reads generally provide lower per-read error rates and are well suited to consensus polishing, whereas long reads can support rapid analysis and resolve longer fragments but require more careful error handling. Hybrid short-read/long-read strategies may improve completeness or confidence in selected settings, but their value depends on viral load, coverage distribution, and the purpose of sequencing [53,54,55].

## 7. Pre-Extraction Enrichment, Virus Concentration, and the Inactivation Boundary

Pre-extraction viral enrichment and virus concentration address a central limitation of random-primed cDNA sequencing: viral nucleic acid may be overwhelmed by host or environmental background. These steps are not separate sequencing strategies but sample processing principles. Depending on the specimen type, they may involve clarification, mechanical disruption, enzymatic treatment, filtration, nuclease treatment, precipitation, particle purification, or related steps that increase the virus-to-host signal before nucleic-acid extraction. The tissue-based universal virus detection for viral metagenomics (TUViD-VM) workflow is an example of this principle in organ tissues [23,24]. It illustrates how strongly upstream sample processing can influence recovery of viral sequence.

For BDBV, the interpretation must be careful. TUViD-VM and related enrichment workflows are conceptually relevant because they improve viral signal in complex specimens, but they are not simple field workflows for suspected filovirus-positive material. They are tissue-oriented, multi-step, equipment-dependent, and may include pre-extraction manipulation that is unsuitable outside appropriate containment for high-consequence filoviruses [23,24].

Virus concentration can improve recovery, but evidence from one matrix or virus cannot be transferred directly to another. Wastewater, respiratory swabs, stool, serum, plasma, tissue, and culture supernatant are not interchangeable. For BDBV, the central question is not whether a method can concentrate virus in principle, but whether it is compatible with clinical specimens, validated inactivation, containment requirements, and field implementation.

Polyethylene glycol (PEG) precipitation is inexpensive and widely used for virus concentration. Comparative wastewater studies show that recovery depends on protocol, matrix, and target virus [25,26]. PEG can perform well for selected low-abundance viral targets, but wastewater evidence does not establish performance for BDBV-positive clinical material. PEG may also co-concentrate inhibitors, and pre-inactivation processing of filovirus-containing specimens would require appropriate containment.

Nanotrap Microbiome Particles (Ceres Nanosciences, Inc., Manassas, VA, USA) and magnetic hydrogel particle approaches are relevant as enrichment concepts [25,67]. Magnetic hydrogel particles improved nanopore sequencing of SARS-CoV-2 and other respiratory viruses from diagnostic remnant swabs by increasing viral mapped reads and improving coverage in lower-titer samples [67]. These approaches are promising for rapid pre-analytical enrichment, but they are not established BDBV clinical sequencing methods.

Apolipoprotein H (ApoH)-based capture is another broad particle-binding concept. ApoH-coated capture has been evaluated for rotavirus detection, and ApoH interactions with hepatitis C virus have been explored as a tool for sensitive viral detection [68,69]. These studies support the principle of broad viral particle capture but do not provide direct evidence for BDBV WGS.

Ultracentrifugation can concentrate or purify virions effectively, but it is poorly suited to decentralized BDBV outbreak sequencing. For filoviruses, virus isolation and concentration of infectious material are maximum containment reference laboratory activities. Their role is reference material generation, phenotyping, assay validation, and countermeasure research, not routine field genome generation.

The critical distinction is whether any enrichment of intact particles occurs before or after validated lysis or inactivation. If intact virions are concentrated before lysis, infectious material may be concentrated as well. If validated lysis and inactivation occur first, subsequent steps are better described as nucleic acid extraction, purification, concentration, or library-level target enrichment rather than intact virus enrichment. This distinction is independent of whether downstream sequencing uses random priming, sequence-independent amplification, probe-based hybrid capture, or tiled amplicon sequencing. Table 3 summarizes sample processing, target enrichment, and sequencing approaches relevant to BDBV-like outbreak settings.

## 8. Probe-Based Hybrid Capture

Probe-based hybrid capture is a target enrichment strategy that uses oligonucleotide probes, or baits, to hybridize to viral sequences of interest. The term “hybrid” refers to this probe–target hybridization step. After hybridization, probe–target complexes are captured and enriched before sequencing. This increases the fraction of viral reads and can improve genome recovery from samples with high host background or low viral RNA input, while generally tolerating more sequence divergence than short primer-dependent tiling schemes [13,14,18].

This category includes broad viral capture systems and probe panel workflows such as the virome capture sequencing platform for vertebrate viruses (VirCapSeq-VERT), ViroCap-type capture, scalable probe sets designed with compact aggregation of targets for comprehensive hybridization (CATCH), the Twist Comprehensive Viral Research Panel, and the Illumina Viral Surveillance Panel [6,13,14,18]. These are target-enriched workflows rather than sequence-agnostic random-primed cDNA workflows, because enrichment depends on the design space of the probe panel. However, they are usually less narrowly target-dependent than tiled amplicon sequencing because longer probes and overlapping panel designs can tolerate more sequence divergence than short PCR primers.

This approach is particularly relevant to BDBV. The 2026 genome report described the use of Illumina RNA Prep with Enrichment and the Twist Comprehensive Viral Research Panel in the DRC and the Illumina Viral Surveillance Panel in Uganda [6]. These are probe-based target enrichment workflows. They are appropriate when the virus family or species is suspected or known and the immediate goal is to recover genomes despite limited prior sequence diversity and high host background.

The main limitations are cost, hands-on time, reagent supply, panel dependence, and possible capture bias. Probe panels can miss viruses outside their design space or under-enrich divergent genomic regions. Bioinformatically, hybrid capture data are usually analyzed by quality control, host read removal, mapping to suitable references, consensus generation, and coverage assessment across the genome. *De novo* assembly may still be useful when divergence is high, but uneven capture efficiency must be considered when interpreting coverage gaps or apparent missing regions.

## 9. Primer-Based Target Enrichment, Tiled Amplicon Sequencing, and Field Deployment

Tiled amplicon sequencing is primer-based target enrichment. It uses multiplex PCR to amplify overlapping genome regions before sequencing. This makes it fast, sensitive, relatively inexpensive, and compatible with both short-read and nanopore platforms once an appropriate tiling primer scheme has been designed and validated. Portable amplicon sequencing during EBOV surveillance demonstrated that WGS can be moved closer to outbreak sites when extraction, amplification, sequencing, analysis, and logistics are integrated into a complete workflow [17,52]. Multiplex amplicon approaches developed for Zika virus and other RNA viruses further established the general utility of primer tiling approaches for direct sequencing from clinical samples [53].

The major weakness is primer dependence. Amplicon sequencing can fail or produce uneven genomes when mutations occur in primer binding sites. SARS-CoV-2 provided a large-scale demonstration of both the power and fragility of tiled amplicon sequencing: primer schemes required updates as variants accumulated mutations, and primer choice affected detection of lineage-defining or diagnostically relevant mutations [15,16,61,62,63,64,65]. Mpox virus provides another useful comparator because an amplicon scheme was rapidly developed in response to an expanding outbreak and compared with broader non-amplicon sequencing workflows [66]. While BDBV belongs to a different virus family with different biology, this workflow development logic is also relevant: once enough viral genomes exist to design and validate primers, tiled amplicon sequencing can become the most scalable option.

For BDBV, tiled amplicon sequencing should therefore not be the only first-line genome recovery approach at outbreak recognition. A tiling scheme designed from sparse historical genomes may fail in divergent regions. Once initial outbreak genomes have been generated by sequence-agnostic random-primed cDNA sequencing, sequence-independent amplification, or probe-based hybrid capture, primer binding sites can be checked and schemes modified. At that stage, tiled amplicon sequencing becomes attractive for rapid, decentralized, lower-cost scale-up.

Amplicon bioinformatics differs from sequence-agnostic or hybrid capture workflows. Primer sequences should be trimmed, amplicon dropout should be monitored, low-coverage regions should be flagged, and consensus generation thresholds should be explicit. Variant interpretation requires particular caution because early-cycle amplification bias, primer mismatch, uneven amplicon coverage, and low input can distort apparent variant frequencies. For outbreak genomics, tiled amplicon data are strongest for consensus genome recovery and lineage tracking after scheme validation; they are weaker for pathogen discovery and for unvalidated intrahost variant analysis.

## 10. Bioinformatics Workflow Consequences of Wet Laboratory Choice

Wet laboratory category and sequencing platform determine the appropriate bioinformatics workflow. Sequence-agnostic random-primed cDNA data require host background subtraction, taxonomic classification, contamination assessment, and *de novo* or reference-guided assembly. Sequence-independent amplified cDNA data require the same analyses plus caution about amplification bias and chimeras. Probe-based hybrid-capture data require coverage bias assessment across probe target regions as well as standard consensus generation. Tiled amplicon data require primer trimming, amplicon dropout monitoring, explicit consensus thresholds, and caution in variant frequency interpretation. Short-read, long-read, and hybrid assembly strategies are therefore not interchangeable analytical afterthoughts but part of the WGS workflow design. SARS-CoV-2 genomic surveillance comparisons, including platform-specific and hybrid assembly analyses, provide useful implementation lessons for outbreak settings even though the pathogen, biosafety constraints, and target diversity differ from BDBV [54,55].

## 11. Inactivation, Biosafety, and Sample Processing Constraints

For high-consequence RNA viruses, inactivation is a workflow determinant rather than a procedural detail. Sequencing from validated inactivated material is fundamentally different from concentrating, culturing, or purifying infectious virus. Published inactivation studies show that assumptions based on generic “lysis buffer” terminology are not sufficient. Inactivation depends on reagent composition, virus, matrix, volume, incubation time, and downstream workflow [56,57,58,59,60,70,71,72].

Some commonly used lysis buffers have failed to fully inactivate viruses under tested conditions [56]. TRIzol LS Reagent (Invitrogen/Thermo Fisher Scientific, Waltham, MA, USA) has been evaluated as a broad viral inactivation reagent under defined conditions [57]. Buffer AVL (QIAGEN GmbH, Hilden, Germany) alone did not inactivate EBOV in a representative clinical sample type, whereas other chemical inactivation workflows have been evaluated for EBOV and related high-consequence viruses [58,59]. More recent work has focused on inactivation methods compatible with downstream sequencing or on validation of Buffer AVL/ethanol-treated material for Ebola, Marburg, and Lassa viruses [60,70]. Broader inactivation studies from other viral systems further emphasize that validation must be workflow-specific [71,72].

For BDBV outbreak WGS, the practical implication is that field or regional workflows should preferably start with validated inactivation and RNA extraction before downstream library preparation. After RNA extraction, the sequencing strategy should be chosen according to the diagnostic and epidemiological question. Pre-inactivation concentration of intact virions may improve recovery in theory but increases the containment and implementation requirements to levels that are not feasible under field conditions or in laboratory settings below fully equipped reference laboratories.

## 12. Culture-Derived Sequencing

Culture-derived sequencing has an important but limited role. Virus isolation can generate abundant viral RNA and high-quality reference material. It is useful for assay validation, phenotyping, neutralization studies, antiviral testing, and countermeasure development. However, for high-consequence RNA pathogens, such as BDBV, isolation of infectious virus and downstream concentration require maximum containment infrastructure.

Culture-derived genomes should not be treated as equivalent to direct clinical genomes for outbreak reconstruction. Culture adds time, may select culture-associated changes, and can create duplicate or replicate sequences that inflate apparent sampling if not annotated and dereplicated. This is especially important for BDBV because the historical genome dataset is small and includes several sequences with different passage histories or technical origins that stem from single clinical samples [2,3].

For real-time outbreak response, direct sequencing from inactivated clinical specimens is preferable for species confirmation, diagnostic target assessment, and genomic epidemiology. Culture-derived sequencing remains valuable for reference and experimental questions, but it should be separated analytically from direct clinical WGS.

## 13. Data Interpretation: Sampling Bias, Phylogeography, and Countermeasure Relevance

Sparse outbreak genome datasets are vulnerable to overinterpretation. Early genomes often come from accessible locations, referral centers, severe cases, or specimens with high viral load. These genomes are invaluable for species confirmation but may not represent the full outbreak. The 2026 BDBV genome report explicitly cautioned that early timing estimates were Bunia-focused because most genomes still came from Bunia or ex-Bunia cases [6].

For large-scale genomic surveillance, interpretation also depends on standardized visualization, lineage assignment, and metadata-linked reporting frameworks. Tools and nomenclature systems such as Nextstrain and Pangolin illustrate how genome data can be translated into public health interpretation when sampling density, metadata quality, and analytical assumptions are explicit [73,74]. For BDBV, comparable frameworks would need to remain cautious because available genome numbers are still sparse and geographically uneven.

Dereplication is essential. Multiple sequences from the same patient, isolate, culture passage, or technical replicate should not be treated as independent biological samples. In small datasets, pseudoreplication can distort phylogenetic structure, apparent diversity, and introduction inference [1,2,3,6].

Intrahost variants require additional caution. Deep sequencing can identify within-host variation, but interpretation depends on coverage, viral load, library method, sequencing platform, replicate support, and error correction [7,8]. Amplicon sequencing and sequence-independent amplification can distort variant frequencies. Intrahost variants should not be used for transmission inference unless the workflow is validated for that purpose.

Phenotypic interpretation should remain conservative. Sequence changes in GP, polymerase, or diagnostic target regions can generate hypotheses about assay performance, antibody binding, vaccine relevance, or antiviral susceptibility. They do not prove biological effect without experimental validation [8,40,41,42,43]. Table 4 summarizes the categories of evidence used to interpret workflow relevance for BDBV-like outbreaks.

## 14. Recommended Staged WGS Strategy for BDBV-like Outbreaks

**Figure 1:**
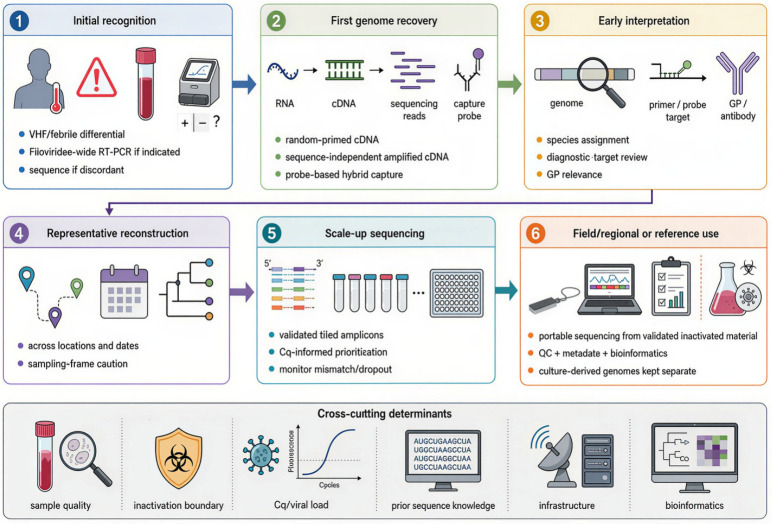
Staged whole-genome sequencing strategy selection for high-consequence emerging RNA viruses under BVD-like outbreak constraints. The schematic summarizes the transition from diagnostic recognition and first genome recovery to representative reconstruction, scale-up sequencing, decentralized implementation, and reference characterization. Abbreviations: BVD: Bundibugyo virus disease; cDNA: complementary DNA; Cq: quantification cycle; GP: glyscoprotein; QC: quality control; RNA: ribonucleic acid; RT-PCR: reverse transcription polymerase chain reaction; VHF: viral hemorrhagic fever.

A single recommended protocol is less useful than a staged strategy (Figure 1, Table 5). The stages should be defined by the outbreak question, sample constraints, and available sequence information, not by a fixed EBOV-first diagnostic pathway.

During initial recognition, the priority is diagnostic breadth, not pan-filovirus testing alone. Because the clinical presentation of filovirus disease is non-specific and overlaps with other viral hemorrhagic fevers, malaria, and other tropical febrile illnesses, suspected BDBV-like outbreaks require a VHF and febrile illness differential front end. Filoviridae-wide RT-PCR, species-specific ebolavirus and marburgvirus testing, CCHF testing, malaria diagnostics, and broader sequence-agnostic cDNA sequencing or discovery sequencing should be selected according to clinical context, epidemiology, local pathogen ecology, and available material [27,28,29,30,31,32,33,34,35,36,37,38]. EBOV-focused negative results should not close investigation when clinical and epidemiological suspicion remains compatible with filovirus disease; expanded differential testing or sequence-agnostic genome recovery remains mandatory when the causative agent is still unclear.

During first genome generation, the priority is sequence divergence tolerance and recovery from available clinical material. Sequence-agnostic random-primed cDNA sequencing may be appropriate when the diagnostic differential remains open or when targeted assays fail. Sequence-independent amplification can help with low-input or sequence-uncertain material but requires caution because amplification bias can distort coverage and variant frequencies [21,22]. Probe-based hybrid capture is particularly suitable when the virus family or species is suspected but tiling primer compatibility is uncertain [6,13,14,18].

During early reconstruction, the priority is representative sampling. Genomes should be generated across locations, dates, clinical contexts, and plausible transmission chains. Early tMRCA and phylogeographic estimates should be explicitly limited to the sampling frame [3,6].

During scale-up, validated tiled amplicon sequencing becomes attractive. Initial outbreak genomes should be used to assess primer compatibility, update tiling primer schemes, and monitor dropout [15,16,17,52,53,61,62,63,64,65,66]. Quantification cycle (Cq) values from diagnostic RT-qPCR can help prioritize samples likely to yield interpretable genomes, as SARS-CoV-2 genomic surveillance guidance and benchmarking studies showed that high viral load and low cycle threshold (Ct)/Cq values improve genome recovery [75,76,77,78,79]. However, Cq-based sequencing triage must not narrow diagnostic screening to BDBV-specific RT-qPCR alone; every epidemiologically relevant specimen should retain sufficient diagnostic breadth to detect EBOV, Sudan virus (SUDV), Marburg virus (MARV), Crimean–Congo hemorrhagic fever virus, or unexpected agents when clinically plausible.

During decentralized surveillance, portable sequencing can reduce delay, but only if the full workflow is supported. Local sequencing without robust quality control, metadata, and analysis may produce genomes that are difficult to interpret [17,52,53].

During reference characterization, culture-derived sequencing and deeper experimental work can support countermeasure research. These data should remain analytically distinct from direct clinical genomes used for outbreak reconstruction [40,41,42,43].

## 15. Future Directions

Several priorities follow from the BDBV case example.

First, BDBV-compatible outbreak algorithms should avoid EBOV-specific testing as a gatekeeper and should not treat pan-filovirus testing as the whole front end. Initial recognition should combine VHF and febrile illness differential testing with Filoviridae-wide diagnostics where clinically and epidemiologically indicated.

Second, probe-based hybrid capture WGS should be strengthened as an early response option for under-sampled high-consequence RNA viruses. This is a practical target enrichment compromise when the virus family is suspected but tiled primer compatibility is uncertain.

Third, tiled amplicon schemes should be developed for BDBV but deployed with caution. Primer binding sites should be checked against current outbreak genomes, and dropout should be monitored continuously.

Fourth, representative sampling should be treated as a response priority. A few early genomes from one location are sufficient for species confirmation but insufficient for robust phylogeography, timing, or transmission reconstruction.

Fifth, decentralized sequencing should be built around validated inactivation, extraction, quality control, metadata capture, and bioinformatics. Sequencing devices are useful only when the surrounding laboratory and analytical systems are functional.

Sixth, data interpretation should separate direct clinical genomes, culture-derived sequences, technical replicates, and low-quality genomes. Small datasets are especially vulnerable to pseudoreplication and overinterpretation.

## 16. Conclusions

The 2026 BVD outbreak illustrates the central challenge of WGS under outbreak constraints. BDBV was known before 2026, but sparse historical genomic sampling, EBOV-centered diagnostic assumptions, high biosafety requirements, and resource-limited outbreak conditions made rapid and representative genome generation operationally difficult.

Sequence-agnostic random-primed cDNA sequencing is valuable during diagnostic uncertainty but can be inefficient in high-host-background samples. Pre-extraction viral enrichment can improve viral signal but may be difficult to implement for high-consequence RNA viruses outside specialized containment if intact particles are concentrated before validated inactivation. Probe-based hybrid capture offers a strong early response option because it improves sensitivity while preserving greater sequence divergence tolerance than narrow tiled amplicon schemes. Tiled amplicon sequencing is best suited for scale-up once outbreak genomes are available and primer compatibility has been validated. Portable sequencing can reduce turnaround time, but only when embedded in a complete workflow, including bioinformatics appropriate to the wet laboratory method and sequencing platform.

The main lesson is that outbreak genomics is a constrained implementation problem. Useful genomes must be generated quickly enough to inform response, safely enough considering the pathogen and setting, broadly enough to tolerate sequence uncertainty, and representatively enough to support diagnostic, countermeasure, and phylogeographic interpretation. For BDBV and comparable high-consequence RNA virus outbreaks, WGS should be staged, adaptive, and explicitly linked to operational realities.

## Figures and Tables

**Table 1:** Outbreak questions and sequencing requirements.

Outbreak Question	Required Information	Suitable Approaches	Main Limitation If Inadequate
Which virus species is causing disease?	Species-level sequence assignment	VHF and febrile illness differential testing, Filoviridae-wide RT-PCR where indicated, broad RT-PCR product sequencing, random-primed cDNA sequencing with taxonomic classification and/or *de novo* assembly, probe-based hybrid capture, WGS	Narrow species-specific negative results may delay recognition of BDBV, other filoviruses, CCHF, malaria, or unexpected agents
Are diagnostic assays compatible?	Sequences across primer and probe targets	WGS, probe-based hybrid capture, or sequencing of assay target regions	Reduced sensitivity or false-negative assumptions
Are GP-dependent countermeasures plausibly relevant?	GP sequence across conserved and variable antigenic regions	WGS, GP-targeted sequencing, or probe-based hybrid capture	Unsupported extrapolation from EBOV, SUDV or historical BDBV
Is spread local, cross-border, or multi-focal?	Representative genomes across time and geography	Scaled WGS linked to metadata	Misleading phylogeographic or tMRCA inference
Can sequencing be decentralized?	Reliable genomes from local or regional workflows	Portable nanopore sequencing, regional short-read sequencing	Delays from transport to distant reference laboratories
Are intrahost variants interpretable?	High-depth, replicate-supported variant calls	Deep sequencing with strong controls	Artifacts from amplification, low input, sequencing error, or contamination

Abbreviations: BDBV: Bundibugyo virus; CCHF: Crimean–Congo hemorrhagic fever; cDNA: complementary DNA; EBOV: Ebola virus; GP: glycoprotein; RT-PCR: reverse transcription polymerase chain reaction; SUDV: Sudan virus; tMRCA: time to most recent common ancestor; VHF: viral hemorrhagic fever; WGS: whole-genome sequencing.

**Table 2:** RNA virus WGS workflow categories by prior viral sequence knowledge.

Workflow Category	RT/cDNA or Enrichment Step	Prior Viral Sequence Knowledge	Usefulness and Main Limitation
Direct RNA sequencing	Native RNA; no RT/cDNA conversion	Low in principle	Conceptually sequence-agnostic, but niche for outbreak WGS because of input and platform constraints
Random-primed cDNA sequencing	Random RT of total RNA; no target selection	Very low	Broadest routine cDNA-based discovery/WGS option; inefficient when host background dominates
Background-depleted random-primed cDNA sequencing	Host/rRNA depletion before random-primed cDNA sequencing	Low	Improves non-host read fraction; depletion efficiency is matrix- and workflow-dependent
Particle-enriched random-primed cDNA sequencing	Pre-extraction enrichment of particle-protected nucleic acid	Low	Can increase viral signal; may bias toward intact particles and is containment-sensitive for filoviruses
Sequence-independent amplified cDNA sequencing	Tagged random primers plus universal amplification; SISPA-like	Very low	Useful for low input or sequence uncertainty; adds amplification bias, uneven coverage, and possible chimeras
Probe-based hybrid capture	Random-primed cDNA followed by viral probe capture	Moderate	Improves viral read fraction and tolerates some divergence; panel-dependent and potentially capture-biased
Tiled amplicon sequencing	Genome-spanning multiplex PCR after RT	High	Fast, sensitive, and scalable after validation; vulnerable to primer mismatch and amplicon dropout
Targeted short-region Sanger sequencing	Gene-specific RT-PCR or PCR for one/few loci	High	Useful for confirmation or genotyping; not WGS unless amplicons tile across the whole genome

Abbreviations: cDNA: complementary DNA; PCR: polymerase chain reaction; RNA: ribonucleic acid; rRNA: ribosomal RNA; RT: reverse transcription; RT-PCR: reverse transcription polymerase chain reaction; SISPA: sequence-independent single-primer amplification; WGS: whole-genome sequencing.

**Table 3:** Sample processing, target enrichment, and sequencing approaches relevant to BDBV-like outbreaks.

Method	Evidence Base Considered	Main Advantage	Main Limitation	Interpretation for BDBV-like Outbreaks
TUViD-VM/pre-extraction enrichment	Tissue metagenomics and pre-extraction enrichment workflow [23,24]	Strong enrichment from complex tissue	Multi-step, equipment-dependent, containment-sensitive	Conceptually important, not a simple field workflow
Ultracentrifugation	Classical virion concentration; used in specialized workflows [23,24]	Strong purification or concentration	Requires specialized equipment and appropriate containment	Reference lab use, not routine decentralized sequencing
PEG precipitation	Wastewater concentration studies [25,26]	Cheap, simple, scalable	Matrix-dependent recovery; inhibitors; biosafety if pre-inactivation	Possible concept; requires BDBV-specific validation
Nanotrap or magnetic hydrogel particles	Wastewater and respiratory sequencing studies [25,67]	Can improve from low-input sequencing samples	Virus- and matrix-dependent	Promising enrichment concept, not BDBV evidence
ApoH capture	Rotavirus and hepatitis C virus capture studies [68,69]	Broad particle-binding principle	Limited outbreak WGS evidence	Conceptual relevance only
Probe-based hybrid capture	Probe-based viral capture and probe design systems [13,14,18]	Increased viral read fraction with broader divergence tolerance than primer-based tiled amplicons	Cost, panel dependence, hands-on time	Strong early response option when suitable probe space exists
Tiled amplicon sequencing	EBOV, SARS-CoV-2, arbovirus, and mpox outbreak sequencing [15,16,17,52,53,61,62,63,64,65,66]	Fast, sensitive, scalable primer-based target enrichment	Primer mismatch and dropout	Strong scale-up option after compatibility checking

Abbreviations: ApoH: apolipoprotein H; BDBV: Bundibugyo virus; EBOV: Ebola virus; PEG: polyethylene glycol; SARS-CoV-2: severe acute respiratory syndrome coronavirus 2; TUViD-VM: tissue-based universal virus detection for viral metagenomics; WGS: whole-genome sequencing.

**Table 4:** Evidence categories for workflow interpretation.

Evidence Category	Examples	Main Value	Main Limitation
BDBV outbreak sequencing	2007–2008 random-primed pyrosequencing plus primer walking, 2012 complete genomes and retrospective sequencing, 2026 probe-based hybrid capture genomes [1,2,3,6]	Most relevant for BDBV targets and phylogeny	Sparse and sometimes geographically biased
Filovirus outbreak sequencing	EBOV outbreak and field WGS [7,8,9,17,44,45,46,47,48,49,52]	Implementation and interpretation lessons	EBOV workflows may not transfer directly to BDBV
Probe-based viral capture systems	VirCapSeq-VERT, ViroCap, scalable probe design [13,14,18]	Increased viral read fraction with broader divergence tolerance than primer-based tiled amplicons	Panel-dependent
Primer-based tiled amplicon sequencing comparators	EBOV, Zika, SARS-CoV-2, and mpox [15,16,17,52,53,61,62,63,64,65,66]	Speed and scale-up lessons	Primer compatibility must be checked
Concentration/enrichment concepts	PEG, Nanotrap, ApoH, TUViD-VM [23,24,25,26,67,68,69]	Improve viral signal in selected matrices	Not BDBV-specific unless validated
Inactivation studies	Lysis buffers, TRIzol LS, AVL/ethanol, chemical inactivation [56,57,58,59,60,70,71,72]	Define what can move out of high containment	Reagent-, matrix-, and workflow-specific
Public health sequencing implementation	National sequencing, short-read, long-read and hybrid assembly, Nextstrain and lineage systems [53,54,55,73,74]	Network and interpretation lessons	Comparator contexts differ from filovirus outbreaks

Abbreviations: ApoH: apolipoprotein H; AVL: Buffer AVL; BDBV: Bundibugyo virus; EBOV: Ebola virus; PEG: polyethylene glycol; SARS-CoV-2: severe acute respiratory syndrome coronavirus 2; TRIzol LS: TRIzol LS Reagent (Invitrogen/Thermo Fisher Scientific, Waltham, MA, USA); TUViD-VM: tissue-based universal virus detection for viral metagenomics; VirCapSeq-VERT: virome capture sequencing platform for vertebrate viruses; WGS: whole-genome sequencing.

**Table 5:** Staged WGS strategy for BDBV-like outbreaks.

Phase	Constraint	Preferred Approach	Main Caveat
Initial recognition	Species/differential uncertainty	VHF/febrile differential; Filoviridae-wide RT-PCR if indicated; sequence when discordant	EBOV-negative result does not exclude BDBV, SUDV, MARV, CCHF, malaria, or unexpected agents
First genomes	Limited material/divergence	Sequence-agnostic random-primed cDNA sequencing, SISPA-like amplification, or probe-based hybrid capture	Match to diagnostic certainty, Cq/viral load, sample quality, panel availability, and discovery need
Early reconstruction	Sampling bias	WGS across locations and dates	Early tMRCA applies only to sampled focus
Scale-up	Many genomes quickly	Validated tiled amplicons with outbreak-compatible tiling primers	Primer mismatch, dropout, and low-diversity sampling bias
Field/regional sequencing	Transport/infrastructure limits	Portable sequencing from validated inactivated material	Requires complete workflow support
Reference characterization	Phenotyping/countermeasures	Culture-derived sequencing and experimental systems	Maximum containment, slower, possible culture adaptation

Abbreviations: BDBV: Bundibugyo virus; CCHF: Crimean–Congo hemorrhagic fever; cDNA: complementary DNA; Cq: quantification cycle; EBOV: Ebola virus; MARV: Marburg virus; RT-PCR: reverse transcription polymerase chain reaction; SISPA: sequence-independent single-primer amplification; SUDV: Sudan virus; tMRCA: time to most recent common ancestor; VHF: viral hemorrhagic fever; WGS: whole-genome sequencing.

## Data Availability

No new data were created or analyzed in this study. Publicly available reports, genome resources, and publications discussed in this review are cited in the reference list.

## Abbreviations

The following abbreviations are used in this manuscript:

ApoH	Apolipoprotein H
AVL	Buffer AVL
BASV	Bas-Congo virus
BDBV	Bundibugyo virus
BSL-4	Biosafety level 4
BVD	Bundibugyo virus disease
CATCH	Compact aggregation of targets for comprehensive hybridization
CCHF	Crimean–Congo hemorrhagic fever
cDNA	Complementary DNA
CHIKV	Chikungunya virus
Cq	Quantification cycle
Ct	Cycle threshold
DENV	Dengue virus
DNA	Deoxyribonucleic acid
DNase	Deoxyribonuclease
DRC	Democratic Republic of the Congo
EBOV	Ebola virus
GP	Glycoprotein
INRB	Institut National de Recherche Biomédicale
L-gene	Large polymerase gene
MARV	Marburg virus
NCBI	National Center for Biotechnology Information
NGS	Next-generation sequencing
NP	Nucleoprotein
oligo-dT	Oligo-deoxythymidine
PCR	Polymerase chain reaction
PEG	Polyethylene glycol
QC	Quality control
RNA	Ribonucleic acid
rRNA	Ribosomal RNA
RT	Reverse transcription
RT-PCR	Reverse transcription polymerase chain reaction
RT-qPCR	Quantitative reverse transcription polymerase chain reaction
SARS-CoV-2	Severe acute respiratory syndrome coronavirus 2
SISPA	Sequence-independent single-primer amplification
SUDV	Sudan virus
tMRCA	Time to most recent common ancestor
TUViD-VM	Tissue-based universal virus detection for viral metagenomics
VHF	Viral hemorrhagic fever
VirCapSeq-VERT	Virome capture sequencing platform for vertebrate viruses
WGS	Whole-genome sequencing
